# An Integrated  Approach for Cancer Survival Prediction Using Data Mining Techniques

**DOI:** 10.1155/2021/6342226

**Published:** 2021-12-28

**Authors:** Ishleen Kaur, M. N. Doja, Tanvir Ahmad, Musheer Ahmad, Amir Hussain, Ahmed Nadeem, Ahmed A. Abd El-Latif

**Affiliations:** ^1^Department of Computer Engineering, Jamia Millia Islamia, New Delhi 110025, India; ^2^School of Computing, Edinburgh Napier University, Merchiston Campus, Edinburgh, Scotland EH10 5DT, UK; ^3^Department of Pharmacology & Toxicology, College of Pharmacy, King Saud University, PO Box 2455, Riyadh 11451, Saudi Arabia; ^4^Department of Mathematics and Computer Science, Faculty of Science, Menoufia University, Shibin El Kom 32511, Egypt

## Abstract

Ovarian cancer is the third most common gynecologic cancers worldwide. Advanced ovarian cancer patients bear a significant mortality rate. Survival estimation is essential for clinicians and patients to understand better and tolerate future outcomes. The present study intends to investigate different survival predictors available for cancer prognosis using data mining techniques. Dataset of 140 advanced ovarian cancer patients containing data from different data profiles (clinical, treatment, and overall life quality) has been collected and used to foresee cancer patients' survival. Attributes from each data profile have been processed accordingly. Clinical data has been prepared corresponding to missing values and outliers. Treatment data including varying time periods were created using sequence mining techniques to identify the treatments given to the patients. And lastly, different comorbidities were combined into a single factor by computing Charlson Comorbidity Index for each patient. After appropriate preprocessing, the integrated dataset is classified using appropriate machine learning algorithms. The proposed integrated model approach gave the highest accuracy of 76.4% using ensemble technique with sequential pattern mining including time intervals of 2 months between treatments. Thus, the treatment sequences and, most importantly, life quality attributes significantly contribute to the survival prediction of cancer patients.

## 1. Introduction

Cancer, along with coronary heart diseases, accounts for most deaths globally (the top 10 causes of death [1]). The incidence rate of cancer has increased over the past few decades. It has been estimated that 1 in 9 Indians will develop cancer during their lifetime. According to GLOBOCAN, India recorded the highest number of deaths globally in ovarian cancer. Ovarian cancer is the third most common site of cancer among women in India. It is also the third most commonly occurring gynecologic cancer worldwide and has the worst mortality rate. Clinicians and scientists have been conducting great experiments and research to predict cancer patients' survivability [2, 3]. Yet, there are no quality survival estimation predictors available. Survival estimation predictors are essential for clinicians to precisely adopt the treatments and medications for the patients.

Data-driven prediction techniques can assist in a better cancer prognosis model. Since its origin, data mining techniques have been efficaciously used in many healthcare research kinds, especially cancer management [4, 5]. The medical models based on data mining techniques can capture intricate details and patterns in data. Several studies involve online datasets like UCI machine learning, SEER [6], and TCGA [7]. However, these datasets only cover datasets from western countries or only from a limited area. Although the number of instances in online datasets is large, these might not capture the region-specific analyses. It has been proven in past studies that race and region can play a significant role in the survivability of cancer patients [8]. Conversely, clinical studies having fewer instances can capture more local aspects of cancer patients and their management. The present research focuses on various attributes that can be significant predictors in estimating the survival of advanced ovarian carcinoma patients that are mostly unavailable in online datasets.

The existing literature focuses on including different clinical attributes like age, CA-125 levels, histology, and stage to investigate the survivability and mortality of ovarian cancer patients [9]. Some of the researchers also intended to explore the outcome for patients treated with neoadjuvant chemotherapy (NACT) [10] or surgery [11]. Nonetheless, the current literature lacks proper research that may give insights into ovarian cancer survival using machine learning approaches and since its initiation, machine learning technology has progressed a lot and is proven to provide good results in almost every area. Some studies performed statistical analysis to find the correlation of treatments with survival [10]. But most of these studies are a part of clinical trials having a controlled environment. A retrospective study in an uncontrolled setting with a variety of participants can point out some useful insights that might not be possible with a clinical trial dataset. Also, to the best of the authors' knowledge, no existing literature emphasized the different sequences of treatments for ovarian cancer patients. Furthermore, various comorbidities can play an essential role in the overall health of patients [12]. The present study involves recording and using some relevant predictors for survival analysis of cancer patients and clinical attributes. These attributes were not available in any online datasets. The collected and processed features can be used and extended to survive any cancer or other serious condition study.

The present study aims to identify the significance of different predictors for advanced ovarian carcinoma patients. An integrated model using attributes from different data profiles can assist in a robust model for predicting survival outcome of patients. The attributes from different data profiles have been collected from a cancer hospital and processed accordingly. Cancer patients are given multiple lines of treatment to prolong their survival. The present study is an attempt to identify the different lines of treatments given to ovarian cancer patients using sequence mining approaches. These treatments and the estimated time elapsed among treatments might contribute some valuable perceptions to the survival of patients. Previous literature has also acknowledged the association of time between treatments with prognosis in patients with ovarian carcinoma [13]. Life quality attributes like performance status and comorbidities also have a significant impact on any person's survival. These attributes have been explored in the study to examine their effect on survival.

The remainder of the study is structured as follows: Section 2 presents a brief background on ovarian cancer and its prevalence in India.  Section 3 provides some of the existing literature on ovarian cancer survival analysis.  Section 4 explains in detail the dataset and the proposed methodology in the study. The study's results and discussion and its comparison to the existing methods are given in Section 5.  Section 6 discusses some of the study's limitations, and the conclusion is presented in Section 7.

## 2. Background

### 2.1. Ovarian Cancer

Ovarian cancer has the worst mortality in all gynecologic cancers. Overweight and obese women have a higher risk of ovarian cancer [14]. Age is also a significant factor in cancer incidence. While its incidence rates have remained constant in some European countries, Asia has experienced increased incidence rates from the past few decades [15]. Survival rates are less than 20% in Indian women. According to a report, 50% of India's total ovarian cancer cases occurred at 45–65 years [16]. Though, most of the western countries have a median range of more than 60 [17].

Ovarian cancer can have around 90% survival rates if detected in the early stage. However, reports have shown that most patients are diagnosed in later stages, with survival less than 40% (SEER Program). This is why it is also known as “silent killer” because more than 60% of the cases are diagnosed at advanced stages (Stages III and IV). Epithelial ovarian cancer is the most common, including high-grade serous, low-grade serous, endometrioid, clear cell, and mucinous. Patients diagnosed in most advanced ovarian cancer cases are provided with multiple lines of treatment. These include cytoreductive surgery (CRS) with adjuvant chemotherapy, or neoadjuvant chemotherapy (NACT) with Interval debulking surgery (IDS) and adjuvant chemotherapy, or hormonal therapy or chemotherapy [18].

### 2.2. Sequence Mining

A sequence ‘seq' is a collection of ordered symbols. |seq| denotes the length of the sequence [19]. A substring of a sequence is a collection of consecutive symbols of the sequence. However, in a subsequence, the symbols need not be consecutive. For example, if PQRS is a sequence with symbols {P, Q, R, S}, then both PQS and PQR can be subsequences of the sequence. But PQS is not a substring of the mentioned sequence. Sequence mining refers to identifying frequently occurring subsequences from a database of sequences. The user determines the term “frequent” by varying the support of the sequences. Support of 0.5 suggests that the database should contain a subsequence in at least 50% of the sequences.

Researchers have devised several sequence mining algorithms. Generalized Sequential Pattern (GSP) [20] is one of the first sequence mining algorithms formulated on the basis of Apriori algorithm [21]. GSP works by identifying the subsequences by scanning the dataset and computing their support. Subsequences with support less than the threshold support are removed from further analysis. For *k* length sequences, GSP scans the dataset *k* times. Once the frequent sequence at level *k* (*k* length sequence) is found, a candidate for length *k* + 1 is generated. Various other researchers also tried to formulate sequence mining algorithms with less time and space complexity. SPADE and PrefixSpan are examples of such algorithms [22, 23].

## 3. Related Work

Several researchers tried to analyze advanced ovarian cancer patients' survival using statistical and conventional survival methods concerning different survival estimators. Vincent et al. [10] used univariate analysis to identify the prognostic factors for stage 3c or 4a ovarian cancer patients. The dataset was collected from 11 French centers and included 483 patients who were treated with NACT followed by surgery. Univariate analysis showed that the absence of cytoreductive surgery (CRS) was associated with worse survival. Similarly, CA-125 value higher or equal to 3000 U/ml had decreased overall survival.

Deng et al. [24] used data from the online dataset SEER (SEER Program) to analyze the survival based on metastatic site for stage 4 patients. Due to the publicly available online dataset, the number of patients is higher than that in other clinical studies. After various inclusions and exclusions, the data analyzed consisted of 1481 patients. Univariate and multivariate analyses showed that the most common sites of metastasis are liver followed by lymph nodes. For patients with lung metastases only, patients who received chemotherapy had a higher survival than those who did not receive chemotherapy. Surgery was also associated with higher survival rates in patients with lymph nodes and liver metastases, but it was not a significant self-determining aspect in patients having lung metastasis. Akhavan et al. [12] also conducted statistical chi squared and Student's test on a dataset collected from Tehran to investigate the effect of diabetes on ovarian cancer survival. The histology considered in particular was epithelial carcinoma. The results suggested that the patients having diabetes had poor overall and progression free survival than those without diabetes.

In a more recent study [25], the authors collected a dataset of around 460 patients from a cancer center to compare white women's survival with black women. The dataset included 365 white patients and 95 black women. It was observed that more white women received surgery, chemotherapy, or surgery chemotherapy sequence. It was also revealed that despite receiving the same treatment sequence, black women had higher mortality rates from ovarian cancer.

Clinicians and researchers from Indian hospitals also conducted statistical tests for survival analysis of advanced epithelial ovarian carcinoma patients. Viswanathan et al. [26] analyzed the data of stage 3 or stage 4 advanced epithelial carcinoma patients diagnosed in years 2015–2018. 111 patients were analyzed by the authors, of which the majorities were of serous histology. Most of the patients were given NACT followed by CRS. It was observed that CRS had improved overall survival and progression-free survival. Also, patients with optimal CRS after NACT had significantly lower recurrence rates and better survival than those suboptimally cytoreduced.

Tseng et al. [27] tried to identify the risk factors in women with ovarian cancer prominent in terms of cancer recurrence. Data mining techniques were used separately using leave one out cross-validation to rank the factors. Since individual data mining techniques cannot address the problem efficiently, the authors used an ensemble approach. The ensemble approach obtained better results than the pure classification techniques, with C5.0 achieving 90% accuracy. Various authors have also used machine learning techniques to predict ovarian cancer. Lu et al. [28] used a decision tree model and feature selection measures to predict the occurrence of ovarian cancer using different blood routine tests, chemistry, and tumor markers. Several other studies also used different classification techniques to predict survival in various types of cancer [29, 30]. However, most of the studies involved online datasets confined to only a specific country or area. While some of those results can be generalized to other regions, it is a well-known fact that cancer behaves differently with different environment and socioeconomic status of the patients [8].

## 4. Methodology

**Figure 1:**
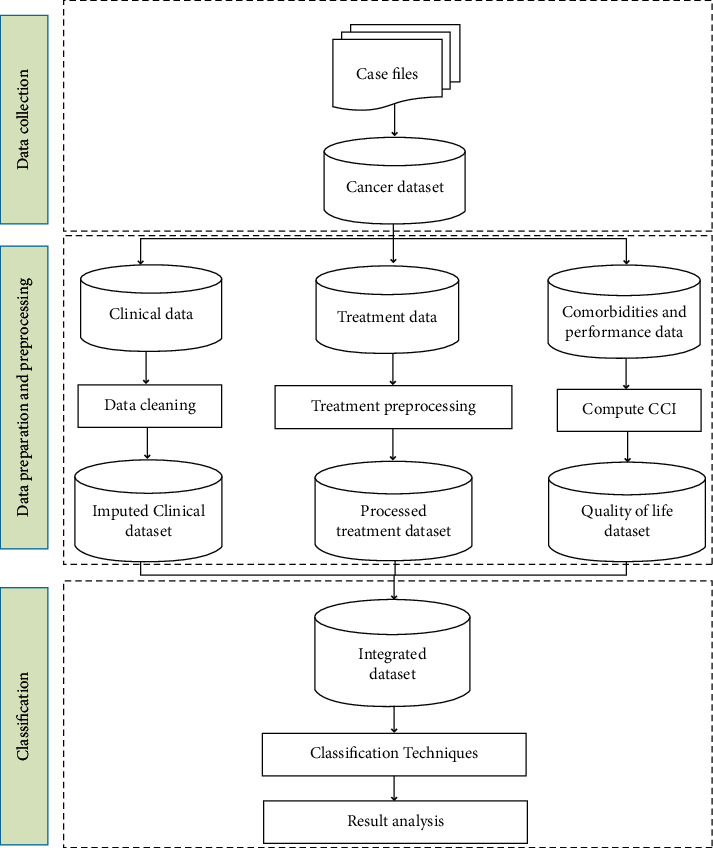
Methodology followed in study.

The proposed methodology of the study is given in Figure 1. This study's approach is divided into three major steps, including data collection, data preprocessing, and classification. The main essence of the study is involved in the dataset used for the analysis. The proposed approach follows an integrated methodology that uses data from three different profiles. However, the medical dataset suffers from many missing and irrelevant data that cannot be directly used for classification. Hence, the second step of the approach involves preprocessing of the dataset according to their data profiles. While clinical data is prepared using standard imputation techniques, we have employed sequence mining techniques to generate treatment sequences given to the patients. Similarly, attributes measuring life quality are created to capture the overall well-being of patients. After all the preprocessing, classification techniques are applied to the integrated dataset. Each step is explained in detail in the following subsections.

### 4.1. Data Collection

This study is based on a dataset collected from a hospital located in New Delhi, India. The case study used for the analysis is of advanced ovarian cancer. The data was collected from the hospital manually from the files digitally stored in the hospital's repository after obtaining appropriate approval from the hospital's Scientific Committee. The study got a waiver from the IRB of the hospital due to anonymity in the use of data. Due to the ethics policies of the hospital, data cannot be shared publicly. The data collected includes three kinds of attributes-clinical attributes, treatment attributes, and comorbidities data. Clinical characteristics including CA-125 levels at the time of diagnosis, presence of ascites, grade, FIGO substage, and histology were collected and recorded for each patient. CA-125 levels denote a diagnostic attribute for ovarian cancer. The presence of ascites and cancer grade define the overall extent and aggressiveness of cancer cells in the body. Higher CA-125 levels, ascites presence, and grade suggest aggressive cancer. Since the collected dataset included advanced cancer patients only, the majority of the patients had stage III or stage IV cancer. Since we have used FIGO substage, stage III cancer patients were further divided into stages 3a, 3b, and 3c cancer. Clinical data has proven to have a high association with the survival and be the most widely used predictors in the existing studies.

Unlike the online datasets and other clinical studies, the present study also collected treatments and appropriate time intervals between each set of treatments given to each patient. The correct treatments given to the patients can prolong their survival. Also, the time elapsed between these treatments might suggest a better or worse response to the treatments. The treatments and the time intervals thus can aid a better survival model. A total of four lines of treatments were recorded for each patient. Most of the patients received less than four treatment lines for three years.

Also, ECOG levels indicating each patient's performance levels were recorded along with several comorbidities like diabetes, heart disease, and hypertension of each patient. The significance of ECOG levels and comorbidities has also been acknowledged in survival analysis of other cancer types [31]. The inclusion of life quality attributes can suggest the overall well-being of the patients and thus can better predict the overall survival of the patients. For a better comparison with existing studies and to include recent and relevant data, patients identified in the years after 2011 and before 2015 were used to collect data. The specified time range also allows for the proper retrieval of survival information of 3 years. Survival of 3 years was collected from the hospital's files or by directly contacting the patient or patient's family.

### 4.2. Data Preprocessing and Analysis

#### 4.2.1. Data Preparation and Preprocessing

All the relevant details and information collected in the previous step were recorded and maintained in a spreadsheet. Each attribute category has been handled accordingly to gain a better perspective and improve patients' overall survival prediction.

*Clinical Data Preprocessing*. Clinical data has been cleaned to remove any outliers and handle missing data. Any instance with missing survival information was removed from the analysis to create a reliable model. Further, instances with more than fifty percent missing data were also removed as larger missing data values can lead to a weak model. The dataset after removing these patients' cases consisted of 149 patients. The rest of the missing data was handled by using mean and mode imputation techniques. Since there were only 9 cases with missing data left and mostly categorical attributes (e.g., presence of ascites), techniques like k-NN imputation did not perform well. Thus, in all the leftover instances, missing numerical attributes were filled out with the mean value of the patients' same class. Similarly, instances with missing nominal attributes were filled with the mode value of the same class. The same has been carried out with MATLAB software using rmmissing() and fillmissing() in-built methods.

**Figure 2:**
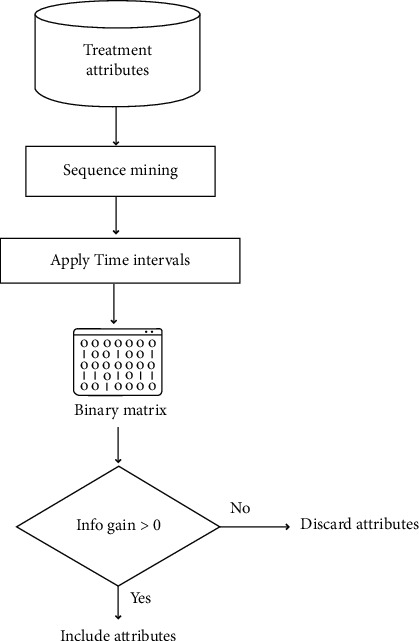
Treatment preprocessing.

*Treatment Data Preprocessing*. The study's objective was to process the data based on each attribute's category and behavior. Treatment preprocessing performed for this study is shown in Figure 2.

The sequences of treatments were processed by creating a database of treatment sequences for each patient. The database generated was supplied to modified sequence mining algorithm GSP. GSP was adjusted to obtain frequent treatment substrings, i.e., treatment sequences with no-gap constraint. The sequence mining algorithm has been implemented in Java [32]. 0.05 value of support has been used for the study to collect the maximum sequences of treatments possible. The no-gap constraint means that intermediate therapies would not be considered for frequent sequences; i.e., if a patient received treatment W in between treatments X and Y, then X ⟶ Y is not a valid recurring sequence. The no-gap constraint is attained at the time of counting support of each candidate sequence. The resultant treatment sequences are mentioned as follows.

**Figure 3:**
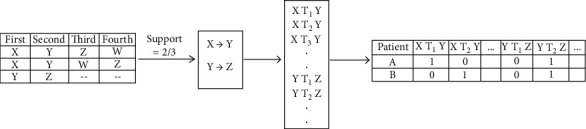
Time intervals in treatment sequences.

The time intervals are applied in the resultant frequent treatment sequences, as shown in Figure 3. The time intervals chosen belonged to the 6 months range, i.e., <=6, 7–12, 13–18, till 31–36. The selection of time intervals was intuitive for 3-year survival and based on the previous literature [33]. Yet, when the data was analyzed, it was observed that most of the patients received their next treatments within 6–8 months of the previous treatment. This may be attributed to the clinical implications of treatments for advanced ovarian cancer patients to commence treatments early [13]. Thus, varying time intervals were chosen (e.g., one month, two months, and three months, till six months) to determine the prognostic value of different time intervals in ovarian cancer survival. A binary matrix is then created based on the attributes. If a patient receives a treatment Y within one month of treatment X, then {X T_1_ Y} column will be marked 1. Since time intervals of 1 month, two months, or three months resulted in 36, 18, or 12 time ranges, it resulted in a wide array of attributes to be applied for classification. Thus, an attribute selection measure was used for the binary matrix. Information gain was computed for each attribute, and attributes having information gain greater than 0 were used for further analysis. The information gain can be calculated using the formula given by the following equations:

(1) $$\begin{array}{c} \text{Info}\left(D\right)=\;-\sum_{i=1}^{m}p_{i}\log_{2}\left(p_{i}\right), \end{array}$$

(2) $$\begin{array}{c} {\text{Info}}_{A}\left(D\right)=\;\sum_{j=1}^{v}\frac{D_{j}}{D}\times I\left(D_{j}\right), \end{array}$$

(3) $$\begin{array}{c} \text{Gain}\left(A\right)=\text{Info}\left(D\right)-{\text{Info}}_{A}\left(D\right). \end{array}$$

*Comorbidity Data Preprocessing*. The comorbidities were collected for each patient as to whether she has a particular condition or not. Comorbidities like chronic obstructive pulmonary disease (COPD), diabetes, hypertension, and coronary artery disease (CAD) were recorded and correspondingly, a metric-CCI was computed for each patient. Charlson Comorbidity Index (CCI) [34] calculates a person's ten-year mortality probability by administering assigned weights to different comorbidities. The higher the computed index, the higher the probability of mortality. For instance, a person having COPD gains +1 score in his/her CCI score. Similarly, patients with uncomplicated diabetes gain an additional +1, while an end organ damaged diabetes gains +3 score their CCI score. Thus, CCI was calculated for each patient to understand the effect of comorbidities better. A summary measure such as CCI is as good as comorbidities used to compute it. Its significance in prognosis has also been proven in the past [35]. Together with the performance status values, CCI constitutes the quality of life part of the dataset in our study. Healthcare based IoT (IoHT) can be further utilized in collecting such life quality data in future studies [36].

#### 4.2.2. Data Summarization and Analysis

The final set of different attributes and their description is shown in Table 1. The final dataset used for the analysis consists of 140 patients with a survival rate of 42.14% (59) and the dataset with a low degree of imbalance does not affect the predictors' performance [37]. Thus, no data balancing techniques have been employed in the study.

**Figure 4:**
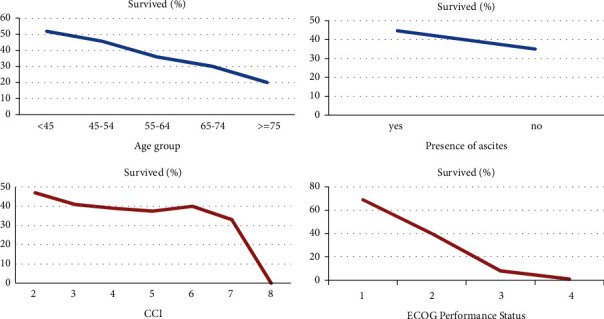
Data analysis with survival.

The present study analyzes survival based on some of the significant attributes and is shown in Figure 4.

Age has always been a controversial factor in the diagnosis and survival outcome for patients. In the present study, it is also revealed that, in younger age groups, patients have better survival outcomes than the older age group patients. However, unlike previous studies [38], ascites' presence has a somewhat opposite effect on advanced ovarian cancer patients' survival outcomes. In our dataset, patients having ascites have slightly better survival than the ones with no ascites present. Nonetheless, the existing literature did not consider the effect of ascites specifically in advanced stage. This result can be further examined by recording and assessing the ascites' volume present in future studies. CCI and ECOG, on the other hand, give promising analyses of survival outcomes. It can be seen from Figure 4 that the higher the values of CCI and ECOG, the lower the survival rate of the patients. ECOG graph shows a sharp declining trend in the chart except at ECOG performance status value 4. This slight change in the graph is that the number of patients with ECOG status 4 was only five, and the survival rate was 0%.

Similarly, a slight increase and inconsistency in the survival rate for patients with CCI score 6 are due to the small percentage of patients in that group. Thus, it is revealed from this consideration that patients with higher age, more comorbidities, and lower level of patient's general functioning are associated with lower survival rate. Other attributes like CA 125, histology, and grade did not show any relevant assessment and were not included in this study.

### 4.3. Classification

The integrated, processed data is supplied to classify the data into survived/deceased class. Ensemble techniques have been successfully used in various medical datasets, and thus their applicability has been tested in the present study. A statistical method, logistic regression, has also been used for comparison with the ensemble approaches.

Bagging and boosting are ensemble classifiers. Bagging or Bootstrap aggregating creates *k* bootstrap sample datasets from the input dataset. Each test instance is classified using various base classifiers, and a combined classifier is created based on each base classifier's votes. The test instance is predicted with the class having majority votes. The averaging factor of voting helps in reducing any kind of variance in the dataset [19]. If the variance of a prediction is *σ*^2^, then the variance of the average of *k* independent predictions is reduced to *σ*^2^/*k*. However, boosting has a weighted average effect. Boosting boosts the performance by giving more importance to instances that are difficult to classify. If a classifier incorrectly classifies an instance, the next classifier provides more significance. Thus, boosting increases that instance's weight. Boosting performs better with weak classifiers as it reduces the bias that could not be removed with bagging. Nevertheless, we may face overfitting in boosting having a weighted approach. In the present study, AdaBoost is a type of boosting algorithm and has been utilized to classify the dataset. Assuming err (**X**_**j**_) to be the misclassification error of tuple *X*_*j*_, then the classifier *M*_*i*_ error rate is the sum of the weights of the misclassified tuples as given in equation (4). The weight of a classifier *M*_*i*_'s vote will be as given in equation (5):

(4) $$\begin{array}{c} \text{error}\left(M_{i}\right)=\;\sum_{j}^{d}w_{j}\times \text{err}\left(X_{j}\right), \end{array}$$

(5) $$\begin{array}{c} \log\frac{1-\text{error}\left(M_{i}\right)}{\text{error}\left(M_{i}\right)}. \end{array}$$

It has been confirmed from the previous studies that ensemble techniques, especially bagging and boosting, can perform better than most of the base classifiers individually.

When the base classifiers used for bagging are all decision tree classifiers, it is known as random forests. The forest denotes the collection of trees into a single unit (combined classifier). Random forest is called random as the decision trees are created using a random selection of attributes to decide the split at each node [39]. Each decision tree votes to determine the class of an instance, and the class with the majority votes is assigned to the test instance [40]. The random forest has proven to give better results in medical datasets. Another popular approach, called XGBoost, has been applied to the dataset using scikit-learn framework. It is a gradient tree boosting approach designed mainly to boost the speed and performance [41]. XGBoost can be used for both classification and regression problems. It is a widely used algorithm by the researchers, specifically for scalable problems [42].

Logistic regression, being a statistical technique, has been used in the present study to compare ensemble techniques with statistical data mining techniques. It models the class membership probability concerning the different attributes of the dataset. It divides the dataset into two classes based on the likelihood of each instance belonging to a particular class. The probability is computed with the help of the attributes' values and estimated coefficients for each feature. The attributes are assumed to be independent to give better performance results. Logistic regression has been used by various authors in different healthcare applications to aid the diagnosis or prediction processes [43, 44].

When some base classifiers like decision trees, SVM, and k-NN were used for the classification process, decision trees gave the best performance for the dataset. The performance of decision trees is acknowledged in various applications due to their high results [45, 46]. Decision trees are also preferred and explored in many medical applications because of their simple and better clarity to the clinicians [47]. Explainable AI is yet another domain that can be explored in future studies. Thus, the classifiers used in this study utilized decision trees for creating the ensemble. Since the dataset used had a small number of instances, 10-fold cross-validation was used for each technique. It divides the dataset into ten equal-sized partitions, where onefold acts as the testing partition, and all the other nine partitions are used for training the classifier. Thus, onefold is treated as a testing partition, and the method is fit on the remaining 9–1-fold. The results on testing partitions of 10-fold cross-validation are averaged. Classification was performed using Classification Learner app on MATLAB software. The final experimental details with highest performance of each classifier used are as illustrated in Table 2.

## 5. Result Analysis and Discussion

**Figure 5:**
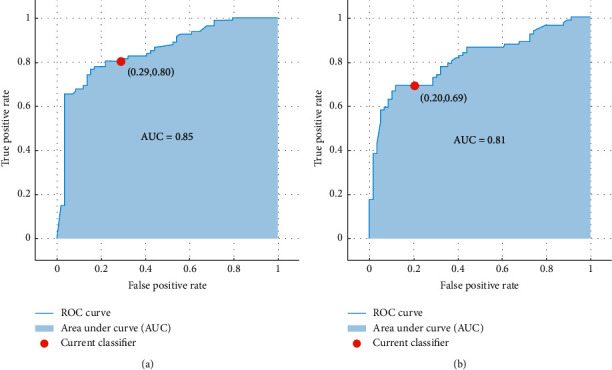
ROC curves for (a) boosting in 2 months' time interval; (b) boosting in 6 months' time interval.

Table 3 shows the results for the time interval sequence mining approach. Time ranges of two months and six months achieved the highest accuracy; thus, Table 3 shows the evaluation measures for only 2 and 6 months. Boosting achieved the best results for both the cases in terms of accuracy and AUC. ROC curves are shown in Figure 5. 5-fold and 15-fold cross-validation techniques were also applied to the dataset to evaluate the methodologies. However, 10-fold gave better results than the other two validation techniques, with the highest of 72.9% for 5-fold and 75.4% for 15-fold. Also, as noted in the previous studies [48], ensemble techniques performed relatively better than the statistical method for our current research as well. However, it can be seen that time intervals of 2 months can better predict the survival of ovarian cancer in almost all the evaluation measures. Six-month approach gave slightly better results in specificity when only boosting is considered.

Contrary to the previous study using six months of time intervals for prostate cancer [33], we have evaluated different time intervals in our study to assess the appropriate time interval for advanced ovarian cancer patients. Thus, time intervals may vary according to cancer type as medications and cancer management differ for each cancer type. An appropriate model may have to be created according to the cancer type and possibly nature of cancer.

The treatment attributes selected for 2 months and 6 months are as given in Table 4. It is further revealed from the Table that the hypothesis in the present study that 6 months' interval might not be useful in the ovarian cancer dataset is true. The attributes selected in 6 months' intervals are only two, with both having T1 (0-6 months) intervals. Conversely, the attributes selected in 2 months' intervals are four and having varying intervals from T1 to T5 only. Thus, it might be possible that only a few, if any, patients received the next line of treatments after say 8–10 months of the previous treatments and, consecutively, did not have any significant role in survival prediction.

Further, each data profile's significance is examined by applying classification techniques to each category of attributes separately for a 2-month time interval integrated dataset.  Table 5 shows the accuracy (in %) along with the classification technique for each data profile.

It is revealed from the results that when an individual category is considered, the life quality dataset performs better than the other data profiles. Also, the treatment dataset gave better accuracy than the clinical dataset. Thus, the treatments' sequences and the time elapsed in the treatments can give the clinicians and patients better knowledge of patients' survival outcomes. This result contributes to the current understanding that, for advanced ovarian carcinoma patients, clinical attributes like CA-125, grade, etc. can indicate selecting the appropriate treatment for the patient. Still, it might not be a good indicator for survival prediction of the patient. Nonetheless, treatment sequences and mostly life quality attributes can be better used in predicting survival outcome.

### 5.1. Comparison of Proposed Work with Existing Literature

**Figure 6:**
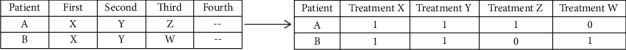
Without sequence treatment processing.

To determine the importance of sequence and time between different therapies given to a patient, we have further compared the proposed approach without sequence mining. A binary matrix has been created for the same, based on the medications received by each patient, irrespective of the sequence in which she received the therapy. An example of such a matrix is shown in Figure 6.

**Figure 7:**
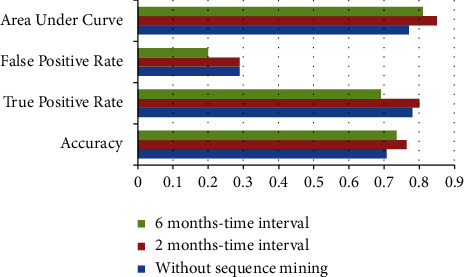
Comparison of results.

The comparison of various evaluation measures for all the approaches is shown in Table 6. Here, time interval approach attained better results than without sequence approach in all the criteria. However, specificity is the same for two-month time interval and no-sequence mining approach. But the overall results improved in the time interval approach. The graphical representation of the results is shown in Figure 7. Also, the random forest gave better results than bagging and boosting in without sequence mining approach. Here, also, the parameter settings for random forest were the same as in the case of proposed approach (i.e., random number seed = 0 and maximum depth = unlimited). Thus, the results for only random forest have been presented in the results. The significance of time in specific treatments has also been acknowledged in previous literature on advanced epithelial ovarian cancer [13]. Hofstetter et al. [49] also demonstrated the use of intervals between surgery and chemotherapy in advanced ovarian cancer patients using statistical techniques. They also revealed that the periods were around 3–6 weeks. The present study also gave better results when time intervals of 2 months were used for the survival prediction. The results were validated statistically by computing *t*-score and corresponding *p*-values with a significance level of 0.05. Since the best results were given by 2-month time interval approach, it was compared with ‘without sequence mining' approach. The results are given in Table 7, and it is evident that the result is significant at *p* < 0.05.

We have additionally generated an assessment of some of the recent studies to compare the data profiles and techniques used in the present study with existing literature, given in Table 8. It can be observed from Table 8 that the majority of the studies used only clinical and treatment data for survival prediction, where treatment data mostly includes details of primary treatment only. Malhotra et al. [56] used treatment sequences along with clinical and genetic data, though the authors did not consider the time elapsed between the treatments. Also, it can be seen from Table 5 that life quality data has a significant contribution to the survival prediction, which is lacking in [56]. The collection and analyses of genetic data, however, can be the future work for the study. Studies using neural networks and deep learning are also becoming more common now with genetic and multimodal data and thus, can be utilized in future studies [62–64]. These have been further explored in various image based datasets as well for the detection and prediction purposes [65–67]. Deep learning technology has been proven in various studies to outperform basic machine learning techniques [68, 69]. However, the dataset in the present study has a smaller number of instances than the existing literature, and deep learning can perform better with large amounts of training data. Due to lack of significant training data, deep learning could not be explored in this study. But this is because the present study involves recent records and only advanced-stage patients. Since earlier stages of almost all cancer already have around 90% survival rates, survival prediction is an easier task. But in the later stages, the survival rates vary from about 10% to 40%. Thus, the present study creates a model established on the cancer behavior (for advanced stage only) that will be more useful for clinicians in examining the survival of cancer patients [70]. It can be observed from Table 8 that almost all the studies used dataset of all the stages. Guo et al. [57] considered earlier cancer patients for the survival prediction and achieved high results. However, as already discussed, earlier stages have considerably higher survival rates and is mostly easier to predict. Thus, more research on advanced cancer patients needs to be conducted to further compare the results.

Thus, it can be concluded from the results that the time interval approach gave better results than no-sequence approach. The time intervals may vary, but the time between treatments can also create a better and reliable predictive model for other cancer patients. The integrated dataset, including data from all profiles, is a better prediction model than the existing models, including only clinical attributes and treatment attributes with no frequent sequences. The clinicians can use this information while deciding the appropriate treatments for advanced ovarian carcinoma patients and the elapsed time between each treatment. The patients' general well-being can also be useful indicators in determining the treatments and corresponding overall survivability of the patients.

## 6. Conclusion

Advanced ovarian carcinoma patients have a poor prognosis compared to early-stage patients. The present study gives some worthwhile comprehensions in advanced ovarian cancer survival. An integrated predictive model has been created using three different data profiles from a real-world clinical dataset. It also focuses on the significance of treatment sequences with varying time elapsed between treatments and various life quality attributes in the survival analysis of patients. Cancer patients are often treated with multiple lines of therapy. The present study validates and ascertains the use of varying time elapsed between treatments in examining the survival of patients using a modified sequential mining algorithm of GSP, and various machine learning techniques. It was revealed that life quality attributes and treatment sequences with the time intervals could predict survival better than clinical facts. Also, time intervals of two months between the treatment sequences performed better than other time intervals with an accuracy of 76.4% and 0.85 AUC. The proposed approach of modified sequential mining algorithm and classification with 76.4% accuracy performed better than the existing approach without sequential mining, giving around 70% accuracy. The results were also statistically validated. Thus, the clinicians and researchers should consider patients' quality of life and line of treatments with time elapsed between them while creating a predictive model for cancer patients.

However, there are a few limitations and possible future aspects worth noting. This study used data from only five years of the hospital to record current medications and other medical technologies. The dataset thus had a small number of instances, which could have resulted in overfitting in classification. Also, the dataset was collected manually by the authors. Some recording errors might have been created in the data. Besides, precise medications and chemotherapy cycles or dosage were not considered to avoid confusion. Yet, these might be useful if we had a larger dataset.

## Figures and Tables

**Table 1:** Dataset description.

	Attribute	Description	Range/values
Clinical attributes	Age	Age at the time of diagnosis	17–80 (median: 54)
	CA-125	CA-125 value at the time of diagnosis	8.7–16301 (median: 929.13)
	Ascites	Presence of ascites in the body	Yes: 114
			No: 26
	Grade	Abnormality level of cancer cells	2–4 (median: 3)
	Stage	Figo substage	3–4 (median: 4)
	Histology	Microscopic regularity of cancer cells	Clear cell: 1
			Endometrioid: 4
			Serous: 111
			Small cell:1
			Germ cell: 1
			Mucinous: 6
			Poorly/undifferentiated: 13
			Mixed: 3

Treatment attributes	Treatment sequences	Frequent treatment sequences obtained after sequence mining	Surgery ⟶ chemotherapy
			NACT ⟶ surgery
			NACT ⟶ hormonal therapy
			Chemotherapy ⟶ hormonal therapy
			Surgery ⟶ hormonal therapy
			Chemotherapy ⟶ CRS
			Surgery ⟶ NACT
Life quality attributes	CCI	Charlson comorbidity index obtained using comorbidities	2–9 (median: 3)
	ECOG performance status	The general well-being of a patient	1–5 (median: 2)

Class attribute	Outcome	Survival outcome after three years of cancer diagnosis	Yes: 59
			No: 81

**Table 2:** Experimental details.

Model	Parameter settings
Bagging	Method = decision trees
	Max number of splits = 139
	Learning rate = 0.1

Boosting	Ensemble method = AdaBoost
	Max number of splits = 20
	Learning rate = 0.1

Random forest	Random number seed = 0
	Maximum depth = unlimited

XGBoost	Maximum number of trees = 100
Logistic regression	—

**Table 3:** Classification results.

		Accuracy (%)	True positive rate or sensitivity	Specificity	Area under curve
6 months	Bagging	71.4	**0.79**	0.61	0.80
	Random forest	70.7	0.64	0.8	0.72
	Boosting	**73.6**	0.69	**0.8**	**0.81**
	Logistic regression	65.7	0.68	0.63	0.70
	XGBoost	71.42	0.71	0.64	0.78

2 months	Bagging	74.3	**0.85**	0.59	0.82
	Random forest	75.7	0.72	**0.81**	0.82
	Boosting	**76.4**	0.80	0.71	**0.85**
	Logistic regression	67.1	0.64	0.71	0.70
	XGBoost	73.8	0.73	0.63	0.79

**Table 4:** Treatment attributes selected.

2 months' time interval		6 months' time interval	
Attributes	Information gain	Attributes	Information gain

Chemotherapy_T5_CRS	0.0458	Chemotherapy_T1_hormonal therapy	0.0408

Surgery_T5_chemotherapy	0.0272	NACT_T1_hormonal therapy	0.008

Chemotherapy_T4_CRS	0.023		
NACT_T1_hormonal therapy	0.01		

**Table 5:** Classification results for each data profile.

Data profile	Highest accuracy in % (classifier)
Clinical dataset	61.4 (bagging)
Treatment dataset	65 (boosting)
Life quality dataset	71.4 (boosting)

**Table 6:** Comparison of results.

	Accuracy	Sensitivity or true positive rate	Specificity	Area under curve
Without sequence mining	0.707	0.78	0.71	0.77
2-month time interval	**0.764**	**0.80**	0.71	**0.85**
6-month time interval	0.736	0.69	**0.8**	0.81

**Table 7:** Statistical significance.

Approach	‘2-months time interval' with ‘without sequence mining'
*t*-value	1.90429
*p* value	0.036491

**Table 8:** Comparison of techniques with previous literature.

S.no.	Authors	Dataset	Type of cancer with stage	Stage of cancer patients used	Type of attributes	Classification technique used	Results
1.	Matsuo et al. [50]	Clinical-768 patients	Cervical cancer	All stage	(i) Clinical	Deep learning and cox proportional model	Mean absolute error of 30.7 (deep learning), 43.6 (cox proportional hazard regression)
					(ii) Treatment		

2.	Park et al. [51]	SEER dataset	Breast cancer	All stage	(i) Clinical	Subgroup mining	Effective rules generated
					(ii) Treatment		

3.	Simsek et al. [29]	SEER dataset	Breast cancer	All stage	(i) Clinical	ANNs and logistic regression	83.6% (ANNs)
							82.9% (LR) for 5-year survival

4.	Wang et al. [52]	Clinical-1075 patients	Lung cancer	All stage	(i) Clinical	Gaussian bayesian network	*R* ^2^ of 93.57% (stage-I), 86.83% (stage-II), 67.22% (stage-III), 52.94% (stage-IV)
					(ii) Treatment		
					(iii) Comorbidities		

5.	García-Laencina et al. [53]	Clinical-399 patients	Breast cancer	All stage	(i) Clinical	KNN, logistic regression, decision trees, support vector machine	81% (highest in KNN)
					(ii) Treatment		

6.	Toth et al. [54]	National health database-28817 patients	Colon cancer	All stage	(i) Treatment	Sequence mining	—
7.	Koo et al. [30]	Clinical-7267 patients	Prostate cancer	All stage	(i) Clinical	Artificial neural networks	84.9% overall 5-year survival
					(ii) Treatment		

8.	Kate and Nadig [55]	SEER dataset	Breast cancer	All stage	(i) Clinical	Logistic regression, naïve bayes, decision tree	84.2% (naïve bayes)
					(ii) Treatment		

9.	Malhotra et al. [56]	Clinical-393 patients	Glioblastoma cancer	All stage	(i) Treatment	Sequence mining with statistical techniques	85% (logistic regression)
					(ii) Genetic		
					(iii) Clinical		

10.	Guo et al. [57]	Clinical-5842 patients	Cervical cancer	Stage IA1 to IIB2	(i) Clinical	SVM, decision tree, random forest, ANN etc.	0.895 and 0.89 AUC (light GBM and random forest)
11.	Kalafi et al. [58]	University Malaya medical cancer registry-8066 patients	Breast cancer	All stage	(i) Clinical	SVM, MLP (multilayer perceptron), decision trees, random forest	88.2% accuracy (MLP)
					(ii) Treatment		

12.	Alabi et al. [59]	SEER dataset	Oral cancer	All stage	(i) Clinical	Logistic regression, SVM, bayes point, boosting, decision forest, decision jungle	88.7% (boosting)
13.	Bos et al. [60]	Clinical-177 patients	Oral cancer	All stage	(i) Clinical	Logistic regression	0.744 AUC
					(ii) Radiomic (MRI)		

14.	Hira et al. [61]	TCGA-579 and 593 samples	Ovarian cancer	All stage	(i) Multi-omics data	Deep learning	93.2–95.5% and 87.1–95.7% accuracy
15.	Proposed approach	Clinical-140 patients	Ovarian cancer	Advanced stage	(i) Clinical	Sequence mining with ensemble	76.4% accuracy and 0.85 AUC (boosting)
					(ii) Treatment		
					(iii) Life quality (comorbidities + ECOG)		

## Data Availability

The study was made possible with the dataset collected from Rajiv Gandhi Cancer Institute & Research Center, New Delhi.
